# Dilute Glycerine

**Published:** 1861-11

**Authors:** 


GLYCERINE, by its strong affinity for water,—acts, in kind, as an escharatic, on raw or mucous surfaces; and some practitioners, for this reason, dilute it one-fourth.

